# Effects of a Diet of *Allium* Extract on Growth, Biochemistry, Metabolism, and Gut Microbiota of Rabbits *(Oryctolagus cuniculus*)

**DOI:** 10.3390/foods13233976

**Published:** 2024-12-09

**Authors:** Antonio Cascajosa-Lira, Silvia Pichardo, Alberto Baños, María Arántzazu Aguinaga-Casañas, Andrea Ricci, Andrea Frabetti, Andrea Barausse, Angeles Jos, Ana M. Cameán

**Affiliations:** 1 Área de Toxicología, Facultad de Farmacia, Universidad de Sevilla, 41012 Seville, Spain; aclira@us.es (A.C.-L.); angelesjos@us.es (A.J.); camean@us.es (A.M.C.); 2 DMC Research Center, Camino de Jayena, 82, 18620 Alhendín, Spain; abarjona@domca.com (A.B.); arancha.aguinaga@domca.com (M.A.A.-C.); andrea.ricci@domca.com (A.R.); 3 Gruppo Martini, 47020 Venice, Italy; a.frabetti@martinigruppo.com (A.F.); a.barausse@martinigruppo.com (A.B.)

**Keywords:** natural additive, organosulfur compound, thiosulfonate, intestinal flora, cuniculture, PTSO

## Abstract

The rabbit farming industry is growing due to the rising demand for healthy, sustainable meat. Rabbit meat’s nutritional benefits and low environmental impact appeal to health-conscious consumers. To enhance economic sustainability, efforts focus on reducing disease susceptibility and antibiotic use through improved biosecurity and natural additives, such as organosulphur compounds from *Allium* plants, which have shown promise in studies for boosting productivity and health. This study aimed to investigate the effects of PTSO supplementation on farm rabbits. Over and after a 76-day period, various parameters were assessed to measure the impacts on rabbit growth, health, biochemical parameters, muscle metabolism, and intestinal microbiota. The rabbit groups received either a control diet or a diet supplemented with *Allium* extract. The results showed significant improvements in growth performance for rabbits fed with *Allium* extract, including higher final weights, increased average daily gain (ADG), and lower mortality rates. A biochemical analysis revealed normal values for the parameters measured in the treated group. A muscle analysis evidenced the presence of a few metabolites of PTSO. A gut microbiota analysis indicated distinct differences between the control and treated groups, increasing the presence of some strains that can influence positively the growth of rabbits. This study highlights the potential benefits of PTSO supplementation for improving growth performance, health parameters, and gut microbiota composition in farm rabbits, suggesting its efficacy as a dietary additive.

## 1. Introduction

The rabbit farming industry is experiencing significant growth, driven by the growing demand for healthy and sustainable meat options. Global rabbit production has been increasing by an average of 1.1% annually over the past decade, with notable growth observed in Africa. Key producers such as China, Spain, Italy, Egypt, and France collectively contribute to more than half of the world’s rabbit meat production [1]. Rabbit meat, known for its excellent nutritional profile, offers high-quality protein, low fat, and a high proportion of unsaturated fatty acids, along with low levels of cholesterol and sodium. These attributes make it an attractive choice for health-conscious consumers seeking alternatives to traditional meats like pork and beef [2,3]. Moreover, rabbit farming is considered more sustainable compared to other livestock industries. It requires less water and feed and produces less greenhouse gas emissions. This makes rabbit meat production an environmentally friendly option, aligning well with the global push towards sustainable agriculture [4].

Over the last decade, the feed efficiency on rabbit farms has been greatly enhanced through progress in genetics, management, and feeding techniques [5]. However, additional improvements are necessary to ensure the economic sustainability of these farms. Rabbits are highly susceptible to various diseases that significantly impact productivity. Additionally, there is a growing demand to reduce antibiotic use in rabbit farming due to consumer health concerns and regulatory pressures. This situation calls for alternative solutions, such as improved biosecurity measures, and enhanced disease prevention strategies, including the use of functional additives that improve animal health [6,7]. In this sense, several supplements included in the diet of rabbits have proved to be effective in improving the growth performance and nutrient digestibility of the animals [8,9,10,11,12,13].

In this regard, edible *Allium* plants, such as garlic (*Allium sativum*) or onions (*Allium cepa*), serve as prominent sources of organosulfur compounds (OSCs), which exert antioxidant, antitumoral, anti-inflammatory, and antibacterial properties [14,15,16,17,18]. *Allium* plant extracts are well-known for their properties that modulate gut microbiota, increasing the beneficial bacterial populations in animal models [19]. Because of these attributes, OSCs have emerged as a potential natural additive suitable for application in nutrition, particularly in animal breeding. Hence, phytogenic compounds derived from *Allium* species show great promise as feed additives. Specifically, propyl thiosulfinate (PTS) and propyl thiosulfonate (PTSO) have been described as being antioxidant, antimicrobial, antifungal, antiparasitic, and anti-inflammatory [20,21]. In the last decade, there has been a notable surge in patents and research investigating the application of PTSO and its analogs as a technological additive. The efficacy of both PTSO and PTS has been validated for the prevention and reduction of parasites in aquatic animals. These substances have played a role in alleviating the residues produced by antiparasitics and antibiotics, thereby contributing to environmental preservation, as highlighted by Baños-Arjona et al. [21]. Previous studies have evidenced the safety of PTSO. In this sense, a lack of toxic effects was reported in genotoxicity studies [22] and a subchronic toxicological study, both in rats [23] and multigenerational studies in mice [24].

PTSO has already demonstrated in various studies the potential to enhance productivity and modulate microbiota in other monogastric species like poultry and pigs [25,26]. Given these beneficial effects in animals, the present study aimed to assess, for the first time, the effect of supplementation with PTSO on farm rabbits. Different hematological and biochemical parameters were measured to determine the efficiency and growth of rabbits after 76 days of exposure to PTSO. Moreover, the metabolites present in the muscle and the effects on the gut microbiota were studied.

## 2. Materials and Methods

### 2.1. Preparation of Experimental Diet and Study Design

The trial was conducted at the Martini Gruppo’s rabbit farm located in Perugia (Umbría, Italy). A commercial line of white Italian rabbits (Oryctolagus cuniculus), genetically selected from the MARTINI strain and of mixed gender, was used in this study. The experiment comprised four batches, each containing 5591 ± 47.69 rabbits. All of the rabbits were sourced from the same season but from different areas within the farm. Two batches were randomly assigned to the control group, and the remaining two batches were assigned to the *Allium* treatment group. A basal pelleted feed, manufactured by the Martini Group, was used as a control diet. The experimental diet consisted of the same feed supplemented with 1 kg per ton of *Allium* extract (Garlicon^®^, DOMCA, Granada, Spain), a flavoring additive standardized to 3% in organosulfur compounds, including thiosulfinates and thiosulfonates. Once the feed was formulated, the active ingredient content was quantified using UHPLC-ESI-MS/MS (Thermo Fisher Scientific, Waltham, MA, USA). The analysis employed PTSO as the reference molecule, yielding a quantified concentration of 27 ± 0.45 mg/kg of feed. This measurement confirmed the presence of the active compounds, ensuring the traceability of the additive within the feed (Figure S1). The detailed composition of both diets is provided in Table 1.

The animals were fed ad libitum from weaning (38 to 55 days) through fattening (56 to 76 days). The ratio of feed to water provided to the rabbits was 1:1.7. The rabbits were housed in rows of cages (8 rabbits per cage) under controlled conditions for ventilation, temperature (19 + 2 °C), and relative humidity (70% to 80%) to ensure an optimal environment for their welfare. The housing facilities were maintained with a minimum light intensity of 20 lux, using both natural and supplemental artificial lighting for at least 8 h per day. A 24 h light/dark cycle was followed, including an uninterrupted 8 h dark period to allow for adequate rest and nocturnal activity.

For the slaughtering of the rabbits, a stunning method followed by rapid exsanguination was employed [27]. All procedures involving animals were performed in accordance with the ethical standards of the institution at which the studies were conducted. The procedures were carried out under veterinary supervision and in accordance with Sanitary Activity Authorization number 02/2013.

### 2.2. Growth Performance

The production parameters recorded were age at weaning, initial number of rabbits, initial weight per rabbit (IWR), initial total weight per batch (ITWB), age at sacrificed (AS), days consuming the diets (DCD), final number of rabbits (FNR), final weight per rabbit (FWR), final total weight per batch (FTWB), average daily gain (ADG), total gain per batch (TGB), mortality percentage (M%), average daily intake (ADI), and feed conversion ratio (FCR). The body weight of the rabbits was recorded at the beginning of the experiment and daily during the 76-day experimental period. For these measurements, the rabbits were weighed using a calibrated electronic scale (Gibertini PTF 7500-C, Milan, Italy). The weights were recorded at the same time of day to minimize potential diurnal variations. The daily feed intake per rabbit was calculated by dividing the total feed consumed by each batch by the number of rabbits in that batch. Feed intake was calculated at the group level and adjusted for the number of animals in each batch to provide an average daily intake per rabbit. The feed conversion ratio was calculated based on the relationship between feed intake and body weight gain according to Elvy et al. [28] and Rahnma et al. [29], using the following formula:

FCR = Total feed intake (g)/Total body weight gain (g)

The FCR was assessed at the end of the 76-day period using the initial and final body weight measurements to determine the total weight gain for each batch.

In addition, once slaughtered, the return of meat weight at slaughter (CWS), total meat return per batch, and carcass yield percentages were evaluated.

### 2.3. Biochemical Blood Indices

Ten blood samples per experimental group were randomly taken under veterinary supervision and sent for analysis to the laboratory medicine service of the Istituto Zooprofilattico Sperimentale delle Venezie (Legnaro, Padua, Italy). The analyzed blood parameters were conducted following standardized procedures and included total protein (g/L), urea (mmol/L), creatinine (µmol/L), glucose (mmol/L), cholesterol (mmol/L), triglycerides (mmol/L), total bilirubin (µmol/L), direct bilirubin (µmol/L), indirect bilirubin (µmol/L), AST (U/L), ALT (U/L), ALP (U/L), GGT (U/L), creatine kinase (U/L), lactate dehydrogenase (U/L), Ca (mmol/L), P (mmol/L), Mg (mmol/L), Na (mmol/L), K (mmol/L), Cl (mmol/L), and Fe (µg/dL).

### 2.4. Metabolism Analysis

#### 2.4.1. Muscle Extraction Procedure

The extraction protocol was performed following the method described by García-Nicolás et al. [30] with some modifications. This method has been previously applied to determine the OSC (PTSO, PTS, s-propylglutation (GSSP), and s-propylcisteine (CSSP)) in the liver, plasma, and urine but not in muscle. Specifically, we have modified the range of concentration assayed, and globally, the conditions were similar to the method applied to the liver. Briefly, 0.2 g of the crushed muscle samples were introduced into a plastic tube. Subsequently, 2 mL of water and 2 mL of acetonitrile (ACN) containing 5% *v*/*v* formic acid (FA) were added. Following vortexing for 1 min, 0.3 g of NaCl were introduced and manually mixed, saturating the aqueous phase and establishing a two-phase system (ACN and water saturated with NaCl) after centrifugation at 10 °C for 5 min at 6000 rpm.

#### 2.4.2. Mass Spectra Analysis and Metabolite Identification

Compounds were analyzed utilizing two different pieces of equipment. On one hand, a Xevo TQ-S micro (Waters) was used to quantify the main component of the *Allium* extract (PTSO) and the derivatives (PTS, GSSP, and CSSP). This piece of equipment consists of a triple quadrupole mass spectrometer equipped with an electrospray I source operated in positive mode and a UPLC BEH C18 1.7 μm (2.1 × 100 mm) column. The injection volume was 5 μL, and the flow rate was 0.4 mL min^−1^. Multiple reaction monitoring was applied where the parent and fragment ions were monitored at Q1 and Q3, respectively. The transitions employed for PTSO were 183.1/42.8, 183.16/76.9, and 183.1/140.9. For PTS, they were 167.1/72.8, 167.1/42.8, and 167.1/124.9. For GSSP, they were 382.1/130.0 and 382.1/50.0, and for CSSP, they were 196.0/106.8 and 196.0/119.9. The first was chosen for quantitation and the others for confirmation. For the UPLC-ESI-MS/MS analyses, the mass spectrometer was set to the following optimized tune parameters: capillary voltage: 2.40 kV, desolvation temperature: 500 °C, source desolvation gas flow: 650 L h^−1^, and source cone gas flow: 50 L h^−1^.

On the other hand, the Thermo Scientific Liquid Chromatography system, which included a binary UHPLC Dionex Ultimate 3000 RS connected to a quadrupole-orbitrap Qexactive hybrid mass spectrometer (ThermoFisher Scientific, USA) equipped with a HESI ionization probe (HESI-II) was used to identify possible metabolites according to Cascajosa-Lira et al. [31].

Extracts from rabbits fed with a normal diet were set as a blank, and a curve with solvent (ACN 50%, 5% formic acid) and a matrix solution were set at the following concentrations: 50–500–1000 ppb of PTSO, PTS, and the known standards metabolites GSSP and CSSP. Furthermore, a validation test was conducted using rabbit muscle containing 500 ppb of PTSO, PTS, GSSSP, and CSSP to ensure the accuracy of the extraction process and confirm the achievement of a suitable recovery. The determination of detection and quantification limits (LOD and LOQ) involved the utilization of the standard deviation (SD) of the response and the slope of the calibration curves. This was achieved through the application of the equations 3xSD/slope and 10xSD/slope for LOD and LOQ, respectively. The SD was computed from the calibration curve by employing the residual standard deviation of the regression line [30].

Metabolite identification was performed following the procedure described in Cascajosa-Lira et al. [31]. Compound Discoverer™ 3.2 (Thermo Fisher Scientific, Waltham, MA, USA) was employed for conducting metabolism studies in both phases I and II.

### 2.5. Gut Microbiota Content

Individual intestinal samples were taken from 10 animals from each group, and a massive sequencing analysis (Illumina) was performed on the total bacterial DNA corresponding to the V4–V5 region of the 16S rRNA gene, which was amplified by PCR. The amplification products obtained were purified using the commercial kit DNA Microbiome Purification Purelink™ (Thermo Fisher Scientific). The amplicons were checked on 1% agarose gels, and the DNA concentration was measured using the Qubit^®^ 3.0 fluorimeter (Invitrogen™, Carlsbad, CA, USA), being normalized to the same concentration in each sample. Bulk sequencing of the amplicons obtained was performed using Nextera XT DNA Library Prep Kit (Illumina, San Diego, CA, USA), obtaining reads 2 × 300 bp in length. Sequencing analyses were performed using the IlluminaMiSeq platform of Novogene Europe (Cambridge, UK).

### 2.6. Statistical Analysis

The data produced from the different batches were statistically analyzed using GraphPad Prism 8 software. Growth performance data were analyzed using a two-way ANOVA, where the independent factors were farm and treatment. As there were no significant differences between farms or replicates and no interaction between farm replicates and treatment, the data were pooled. Subsequently, a *t*-test was performed using the Holm–Sidak method, with a significant level of 95%. The data resulting from the biochemical analysis were subjected to analysis using an unpaired *t*-test or a Mann–Whitney test, depending on whether normality passed the Kolmogorov–Smirnov test or not, respectively. For microbiome data, alpha diversity indices (Shannon index, Chao1 index, Pielou’s evenness, and Good’s coverage) were calculated to assess within-sample diversity. Differences in alpha diversity between groups were evaluated using the Mann–Whitney U test. Beta diversity was assessed using principal coordinates analysis (PCoA) and non-metric multidimensional scaling (NMDS) based on Bray–Curtis dissimilarity matrices to visualize the differences in microbial community composition between groups. A permutational multivariate analysis of variance (PERMANOVA) was employed to test significant differences in beta diversity. Linear discriminant analysis (LDA) effect size was performed to identify differentially abundant taxa between the control and *Allium*-supplemented groups, with a significance threshold of *p* < 0.05 and a logarithmic LDA score > 2.

## 3. Results

### 3.1. Growth Performance

The results obtained in both experimental groups are shown in Table 2. There were no significant differences in the parameters measured at the beginning of the study (n° of rabbits, total weight, and starting weight per rabbit). However, there were significant differences at the end of the trial. The animals fed with Garlicon^®^ showed a significant increase in the total final weight and the total final weight per rabbit. Moreover, the % mortality decreased significantly in the animals fed with *Allium* extract. In addition, the parameters, total gain per batch (TGB), average daily gain (ADG) g/rabbit, and average daily intake (ADI) exhibited a significant increase compared with the control group.

### 3.2. Biochemical Blood Indices

The results of the biochemical analysis are shown in Table 3. The results for most of the parameters measured showed no significant differences with respect to the control group. However, certain variations were observed in the serological levels of cholesterol, gamma-glutamyl transferase (GGT), and potassium (K). Animals fed with *Allium* extract exhibited significantly lower levels of urea, cholesterol, and triglycerides compared to the control group (*p* < 0.01). Additionally, the levels of the enzyme GGT were significantly lower than those in the control group (*p* < 0.05). Lastly, the levels of phosphorus and potassium were significantly different than those in the control group (*p* < 0.05).

### 3.3. Muscle Analysis and Biotransformation Products

After the extraction procedure, the analytical parameters (recoveries, LOD, and LOQ) are presented in Table 4. The calibration curves for every compound obtained in muscle were PTSO: y = 9.1411x + 1497.9; PTS: y = 81.233x + 7804.7; GSSP: 1318.3x + 186841; CSSP: y = 271.97x + 43101.

**Figure 1:**
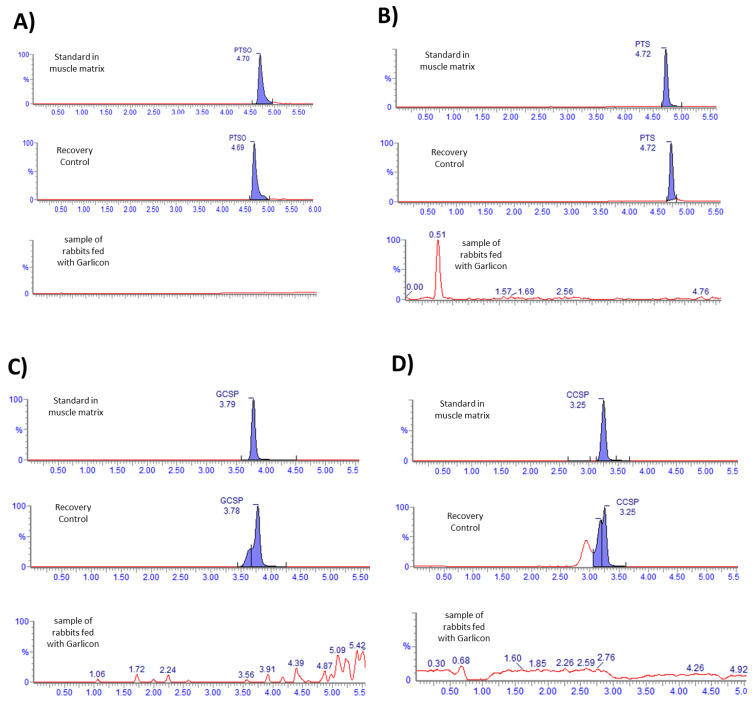
Chromatograms of (**A**) PTSO, (**B**) PTS, (**C**) GSSP, and (**D**) CSSP. Each compound is found in three different situations: the standard in matrix, doped and extracted samples (recovery control), and samples of rabbit muscle fed with *Allium* extract. Retention times are shown for every compound.

The chromatogram profile of every compound is represented in Figure 1. All compounds show a selectivity appropriate to the validated method. The recoveries of all compounds are adequate, being between 88 and 110%. However, none of the compounds were identified in the muscles of the rabbits fed with *Allium* extract.

The metabolites of phase I and phase II detected in the muscles of the rabbits are presented in Table 5. In the muscle tissue of rabbits, a total of nine metabolites have been identified, with the majority arising from phase I reactions, specifically nitro-reduction, followed by desaturation and dehydration reactions. Additionally, phase II reactions are observed, primarily attributed to amino acid conjugation processes.

### 3.4. Gut Microbiota Content

#### 3.4.1. Effect of *Allium* Extract Supplementation on Alpha and Beta Diversity Indices

**Figure 2:**
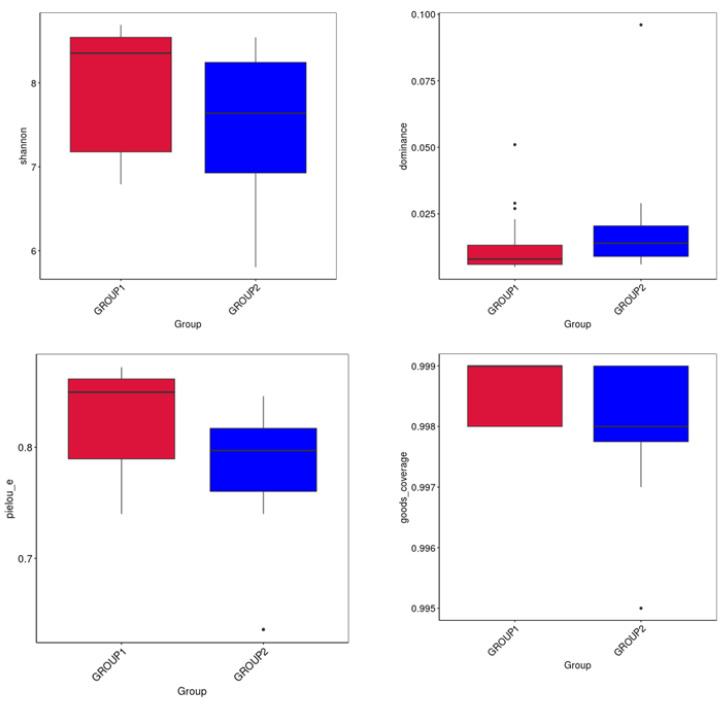
Boxplots of the alpha-diversity indices (Shannon index; Dominance; Pielou’s evenness; and Goods coverage) calculated using specific species abundance data from rabbits fed with Allium extract (Group 1 in red) and control (Group 2 in blue).

Supplementing the diet of rabbits with *Allium* extract affected alpha diversity indices, such as the Shannon index, Chao1 index, Pielou’s evenness index, and Good’s coverage (Figure 2). The *Allium*-supplemented group (Group 1) exhibited a slightly higher Shannon index than the control (Group 2), suggesting greater diversity in terms of species richness. Regarding the Chao index, both groups were similar, with a slight advantage observed in the *Allium*-supplemented group, suggesting it may contain a marginally higher number of unobserved species. A dominance analysis showed significantly lower dominance in the *Allium* group. When analyzing Pielou’s evenness index, rabbits fed with *Allium* extract exhibited a higher value, indicating a more uniform distribution of bacterial species. Both groups displayed high and comparable Good’s coverage. A higher Good’s coverage index implies that a greater proportion of the total species community was sampled, confirming the representativeness of the sample.

**Figure 3:**
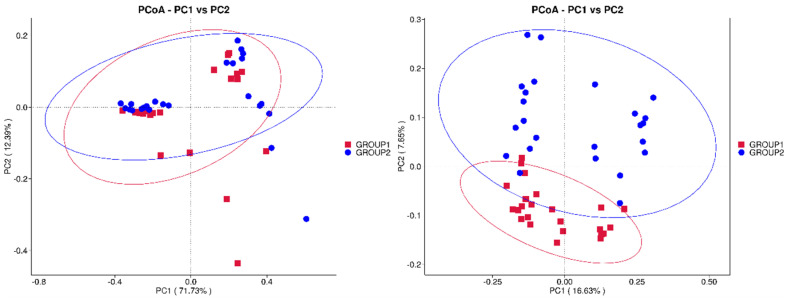
Principal coordinates analysis (PCoA) plots showing the distribution of microbiome samples from rabbits fed with Allium extract (Group 1 in red) and control (Group 2 in blue). The plots represent the first two principal coordinates (PC1 and PC2). The left plot illustrates the overall clustering with PC1 explaining 71.73% and PC2 12.33% of the variance. The right plot focuses on a different variance distribution with PC1 explaining 16.63% and PC2 7.85% of the variance. Ellipses denote 95% confidence intervals for each group, indicating distinct separation in microbial community composition.

A beta diversity analysis was assessed using a principal coordinates analysis (PCoA), as shown in Figure 3. The PCoA plot, based on the first two principal coordinates, revealed a clear separation along the PC1 axis, with significant differences in microbiome composition between the groups. The dispersion ellipses, representing variability within each group, showed limited overlap, reinforcing the distinction between the microbial communities of the *Allium* and control groups.

**Figure 4:**
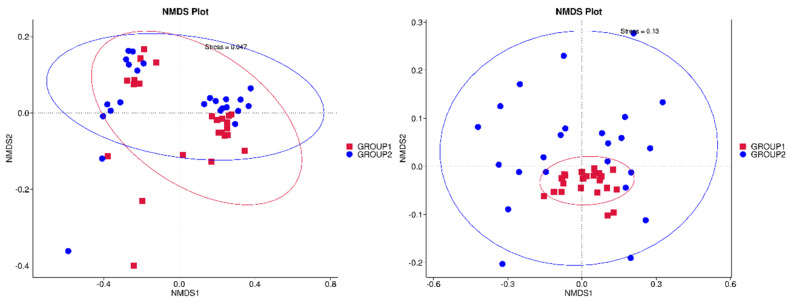
Non-metric multidimensional scaling (NMDS) plots illustrate the microbiome composition of rabbits fed with Allium extract (Group 1 in red) and control (Group 2 in blue). The left plot has a stress value of 0.047, indicating excellent representation in 2D space, while the right plot has a stress value of 0.13, suggesting a good but less precise representation. Ellipses represent 95% confidence intervals, showing distinct clustering and separation between the groups, which reflects significant differences in their microbial communities.

Additionally, NMDS plots were generated to illustrate the distribution of microbiomes between the two groups (Figure 4). The NMDS diagrams revealed significant differences in microbiome composition, with minimal overlap between the *Allium*-supplemented group (red squares) and the control group (blue circles). The *Allium* group showed a more distinct cluster, particularly along the NMDS1 axis. Panel 1, with a stress value of 0.047, provides a highly reliable representation, while Panel 2, with a stress value of 0.13, also shows clear separation. The distinct clustering and consistency between panels confirm robust differences in microbial composition.

#### 3.4.2. Changes in Bacterial Community Composition

**Figure 5:**
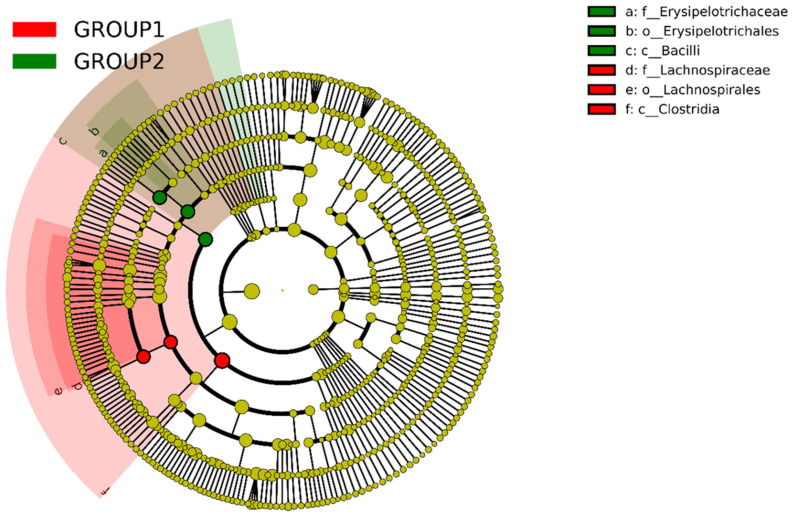
Cladogram illustrating the taxonomic distribution of microbial communities in rabbits fed with Allium extract (Group 1 in red) and control (Group 2 in green). Nodes are colored based on their dominance in each group, with labels indicating taxonomic classifications: (a) f__Erysipelotrichaceae, (b) o__Erysipelotrichales, (c) c__Bacilli, (d) f__Lachnospiraceae, (e) o__Lachnospirales, (f) c__Clostridia.

Significant differences in bacterial community composition were observed between the two groups. Figure 5 represents the cladogram, a graphical representation used to visualize the relationships among different microbial groups in the two groups of rabbits.

The cladogram highlights nodes by their dominance, with red representing the *Allium* group and green representing the control group. The phylogenetic analysis of gut microbiota revealed compositional differences between the *Allium*-supplemented group (Group 1) and the control group (Group 2). Particularly, the *Allium* group exhibited a higher abundance of taxa within the families Lachnospiraceae and the orders Lachnospirales and Clostridia, as indicated by the red nodes and shaded regions. On the other hand, the control group had a greater prevalence of taxa of the families Erysipelotrichaceae, the order Erysipelotrichales, and the class Bacilli, as shown by the green nodes. These findings highlight the significant impact of *Allium* supplementation on the gut microbial community structure, suggesting potential implications for host health and metabolic processes. The minimal overlap between groups underscores the robustness of these dietary effects. 

**Figure 6:**
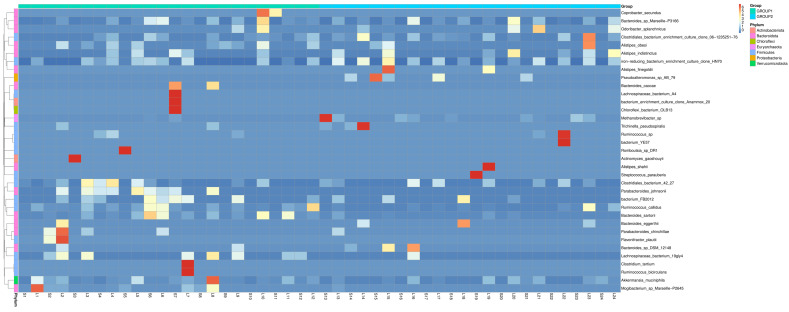
Heatmap showing the relative abundance of bacterial species in rabbits fed with Allium extract (Group 1) and control (Group 2). Columns represent individual samples, and rows represent bacterial species, with color intensity indicating the abundance level (red for high abundance, blue for low abundance). Taxonomic groups are labeled on the right, highlighting differences in microbial composition between the two groups. This visualization underscores the variability and differential abundance of specific bacterial taxa influenced by dietary treatments.

Considering that the different classes comprise both beneficial and potentially pathogenic species, a detailed investigation into species-level abundance changes was necessary. Figure 6 illustrates these changes in a heatmap, highlighting specific variations in the abundance of bacterial species. A significant variability in the presence and abundance of bacteria among samples within each group indicates significant microbiota diversity among individuals in the same group. The bacteria species predominantly found in the *Allium*-supplemented group were *Akkermansia muciniphila* and *Ruminococcus gnavus*, which may be linked to dietary factors.

## 4. Discussion

In the present work, we have studied the beneficial effects of an *Allium* extract on rabbits. The results show that, although there were no differences in the weight and number of rabbits at the beginning of the trial, a higher final weight per rabbit and total weight were obtained for those batches that consumed the *Allium* extract. This fact could be related, on the one hand, to a higher feed consumption of the animals in the *Allium*-supplemented group, which led to a higher average daily gain (ADG), and on the other hand, to a decrease in mortality and the number of losses during the treatment period. It is possible that the increase in consumption could have influenced the decrease in mortality. However, the feed conversion rate was not affected by both treatments. This agrees with the results obtained in other trials in which feed restriction (without any feed supplementation) was carried out in rabbits, improving feed efficiency but negatively affecting mortality [32]. In our trial, the opposite situation occurred, since the animals took an ad libitum diet, observing a decrease in mortality in those that had higher intake but no effect on the feed conversion ratio. These data are similar to those obtained in other monogastric species, where the use of the same *Allium* extract positively influenced productivity. In weaned piglets, the use of this *Allium* extract improves ADG and CI [26]. Similarly, in broilers, it has been observed that the use of PTS-PTSO improved weight gain and the feed conversion ratio [33]. Our results are consistent with those obtained by other authors who observed an increase in intake and ADG in rabbits fed 5 and 10% garlic, as well as a decrease in mortality, probably due to a modulation of immune responses and improvement of the intestinal barrier, and an inhibition of the synthesis of proinflammatory cytokines [34]. In the scientific literature, garlic extracts and oils have been useful for increasing the growth performance in rabbits [35,36,37]. However, all these studies used garlic in combination with other active compounds (nanoselenium and vitamin E) or plants (pepper, anise, thyme, mint…). As far as we know, our study is the first evidence of the usefulness of *Allium* extract standardized with PTSO in the growth of rabbits.

Additionally, the number of rabbits slaughtered tended to be higher in the *Allium*-supplemented group, even though the number of rabbits at the end of the cycle was similar in both groups. It is to be expected that, just as lower mortality was observed in the *Allium* group during the cycle, there may also have been fewer casualties during transport to the slaughterhouse due to an improvement in the general health of the animals. The higher weight at the end of the cycle was reflected in a higher meat return at slaughtering, both per rabbit and in total. However, the yield at sacrifice was similar in both groups, indicating that this difference was due to a greater overall growth of the animal and not to an increase in fat. Similarly, Hernández et al. [38] observed that, in those rabbits with a higher growth rate, the carcass composition is not affected when rabbits are measured at the same stage of maturity. In another study conducted on growing–finishing pigs fed with a diet containing 30 ppm of PTSO, a significant increase in 0–103 d ADG was observed, and the body weight tended to increase at the end of the finishing period compared to animals receiving the control diet. However, at the end of the study, the dietary treatment did not affect backfat thickness or loin thickness in either group [39].

The results for most of the biochemical parameters measured show no significant differences with respect to the control group. However, the results showed a statistical decrease in cholesterol, although these values remain in a normal range [40,41]. Previous work on PTSO and PTS has studied their effect on serum cholesterol levels after exposure in rats for 90 days. Cascajosa-Lira et al. [23] demonstrated a slight decrease in male rats exposed to 55 mg of PTSO/kg/day. However, when the rats were exposed to 55 mg of PTS/kg/day, no effects on total cholesterol, HDL, or LDL were observed [42]. Hence, in this case, the positive impact on cholesterol levels traditionally associated with the bioactive compounds of the *Allium* genus [43] can be attributed to PTSO. Regarding triglycerides, a significant reduction in levels was also observed in the group of rabbits fed with the *Allium* extract. This decrease in triglyceride levels has been previously described in several studies involving both rats and humans that incorporated different garlic or onion extracts into their diets [43,44,45]. The results for gamma-glutamyl transferase (GGT) and potassium (K) showed a significant decrease after exposure to *Allium* extract. Nevertheless, the levels of GGT are still in a normal range for this species [30,39]. Previous studies carried out in Wistar rats using PTSO [23], PTS [41], and PTSO-rich *Allium* extract [22] have shown that these organosulfur compounds do not produce a toxicological alteration in the biochemical levels of enzymes, proteins, lipids, or electrolytes, even when high doses are used. These facts support the results obtained in the present study carried out on a different species such as rabbits.

In relation to the analytical study, it has been applied to a method validated by Garcia-Nicolás et al. [30] in muscle for the first time successfully. The examination of the muscle tissue aimed at identifying the principal components of the tested *Allium* extract (PTSO and PTS) and their associated biotransformation products (GSSP, CSSP), and comprehensive analytical procedures were employed. In addition, this investigative approach sought to elucidate the composition and transformations undergone by PTSO within the muscle matrix. The recovery yields observed in the current investigation, ranging from 88% to 110% for PTSO and its derivatives, align with the values recommended by AOAC [46] and González et al. [47]. Furthermore, the recovery falls within the accepted range of 80% to 110%. No concentrations of PTSO or PTS, nor their known metabolites, such as glutathione conjugated and cysteine conjugated (GSSP and CSSP) were detected in the rabbit muscle fed with *Allium*-supplemented feed. It is important to highlight that these results demonstrate that PTSO does not accumulate in tissues and, therefore, would not affect the sensory properties of rabbit meat. For example, in a similar study conducted on pigs that were also fed a diet supplemented with 30 ppm of PTSO, trained panelists did not find differences in the analyzed attributes compared to the control group. The mean scores of sensory analyses (color, odor, flavor, and juiciness) of cooked meat samples were similar for both the control and the *Allium*-supplemented group [48].

However, some new metabolites were identified. Among them are desaturation and conjugation reactions with amino acids other than those already known, including ornithine, glycine, and glutamine. Recently published studies by Cascajosa-Lira et al. [31] have conducted biotransformation assays utilizing rat and human microsomes. These investigations highlight potential pathways for the biotransformation of PTSO. According to the present findings, PTSO may undergo dehydration followed by reduction reactions (R1), as well as biotransformation involving two consecutive desaturations (R2) and another pathway involving nitroreduction, reduction, and subsequent conjugation with glycine (R6). These pathways are in agreement with the previous metabolites (M8, M3, and M62) found by Cascajosa-Lira et al. [31] using in vitro biotransformation with rat and human microsomes. However, the present study contributes novel insights by identifying previously undisclosed metabolites above mentioned, namely (R3), (R4), (R5), (R7), (R8), and (R9). The novel metabolites in rabbit muscle tissues underscore the significance of amino acid conjugation in the metabolization process of PTSO. Notably, the predominant conjugated amino acids identified in this study were ornithine, glycine, and glutamine. This finding emphasizes the potential role of these specific amino acids in the metabolic pathways involving PTSO. This discovery expands our understanding of the diverse possibilities of biotransformation, shedding light on the variations between species. A recent toxicokinetic study on PTSO in rats [49], conducted following the OECD 417 guideline [50], has identified the metabolites that overlap with those found in the current study in rabbit muscle. The common metabolites are as follows. Metabolite R1 was detected as metabolite T21 in the stomach in the toxicokinetic study conducted by Cascajosa-Lira et al. [49]. Similarly, metabolites R2 and R3 correspond to T37 and T41, respectively, in the stomach from the same study. Metabolite R4 was also identified as T21 in the stomach, while metabolite R9 matches T14, which was found in the liver, kidney, and testes. Conversely, metabolites R5, R6, and R8 were not detected in common with other in vivo studies. This highlights the necessity of investigating metabolite profiles in both laboratory animals and target species, in this case, rabbits, in accordance with EFSA’s Guidance on the Assessment of the Safety of Feed Additives for the Consumer [51]. Additionally, the novel metabolites observed in this study align with phase II biotransformation reactions (e.g., amino acid conjugations), which are known to appear later in exposure [52]. This finding is consistent with the prolonged exposure of 76 days in the current study, compared to the single-dose approach employed in the toxicokinetic study. 

In addition, it is important to highlight that these data demonstrate that PTSO does not accumulate in tissues and, therefore, would not affect the sensory properties of rabbit meat. In a study conducted on pigs fed a diet supplemented with 30 ppm of PTSO, trained panelists did not find differences in the analyzed attributes compared to the control group. Mean scores of sensory analyses (color, odor, flavor, and juiciness) of cooked meat samples were similar for both the control and the *Allium*-supplemented group [48]. Similarly, in the present work, no differences in color or odor were detected upon macroscope examination.

Regarding the gut microbiome analysis, significant differences were observed in both the alpha and beta diversity in the samples from rabbits that were fed with *Allium* extract. The gut microbiota mediates adaptability to the environment and diet in rabbits and provides multiple potential strategies for regulating intestinal health and promoting higher feed efficiency [53]. Therefore, understanding the gut microbiome in meat rabbits is essential for controlling the intestinal microbiota to improve the health and production efficiency of meat rabbits [54]. Supplementing the diet of rabbits with *Allium* extract increased alpha diversity indices, particularly the Shannon index, indicating greater species richness and evenness. The Chao1 and Pielou’s evenness indices were also higher in the *Allium* group, suggesting a more evenly distributed and potentially healthier bacterial ecosystem. A dominance analysis showed lower values in the *Allium* group, indicating a more equitable species distribution and greater microbiome stability. Overall, these results indicate that the group of animals fed with Garlicon^®^ appears to have greater species diversity and consistency compared to the control group, which could be indicative of a healthier microbiome in that group of rabbits [55]. These results are consistent with other studies administering similar *Allium* extracts. For instance, supplementing the diet of gilthead seabream juveniles with PTSO affected the Shannon diversity index in the gut, showing higher diversity compared to the control group [56]. However, these authors using the same *Allium* extract have reported reductions in the alpha diversity index in other species such as seabass and piglets [19]. The beta diversity analysis using PCoA and NMDS demonstrated differences in microbiome composition between the different diet groups, with low overlapping of the microbial communities.

Concerning changes in bacterial community composition, significant differences were also observed between both groups. In the cladograms, the *Allium*-supplemented group showed a significant association with bacteria from the families Lachnospiraceae and Clostridia. The increase of these families in the gut microbiota could have relevant implications. Beneficially, many *Clostridia* and *Lachnospirales* species produce butyrate, a short-chain fatty (SCFA) acid that supports gut health, reduces inflammation, and improves metabolic function [57]. However, some *Clostridia* species can be pathogenic, causing infections and producing toxins that can lead to gut dysbiosis and severe health issues in farm animals [58]. To further investigate, a species-level abundance analysis was performed. Despite considerable variability, the analysis revealed a predominant presence of *Akkermansia muciniphila* and *Ruminococcus gnavus* in the *Allium*-supplemented group. *A. muciniphila* is considered beneficial for gut health due to its role in maintaining the mucosal barrier, reducing inflammation, and improving metabolic functions [59,60,61,62]. In addition, *R. gnavus*, a mucin-degrading bacterium, supports gut health by enhancing immune regulation [63,64] and increasing the production of SCFAs, particularly butyrate, which are crucial for maintaining intestinal integrity and reducing inflammation, as demonstrated in a murine model of atopic dermatitis [65,66]. These SCFAs provide energy to colonocytes, enhance gut barrier function, and have anti-inflammatory effects, contributing to a healthy gut environment and supporting immune function [67]. All these beneficial effects of both strains could explain the improvement in the growth rate of *Allium*-supplemented rabbits. In contrast, no pathogenic species of interest in rabbit farming, such as *Clostridium perfringens* or *Clostridium spiroforme*, were detected in either the control or *Allium* groups [68,69,70,71]. Furthermore, the control group also showed a higher abundance of Bacilli that are associated with improved gut health, highlighting the good sanitary condition of the farm animals [71,72,73,74].

Rabelo-Ruiz et al. [75], in a study conducted on piglets fed a diet supplemented with 20 ppm of PTSO, also reported changes in microbiome composition. In the duodenum and ileum of control piglets, the microbiota was predominantly composed of the classes *Bacilli* and *Clostridia*. The *Allium*-supplemented group exhibited similar patterns but with a reduced proportion of Bacilli and increased proportions of *Gammaproteobacteria* and *Clostridia*. The same research team reported that, after feeding laying hens a diet supplemented with an *Allium* extract rich in PTSO for 30 days, an increase in *Lactococcus* in the ileum and *Lactobacillus* in the *cecum* was observed [75]. Furthermore, in a study performed on mice fed an obesogenic diet, the supplementation with PTSO normalized the gut microbiota composition by reducing Firmicutes and restoring the balance of Bacteroidetes and Verrucomicrobia. This included an increase in *A. muciniphila* and *Lactobacillus*, which is known for its anti-obesity effects and role in maintaining intestinal barrier integrity [19].

## 5. Conclusions

Our study revealed noteworthy outcomes, demonstrating that rabbits fed with *Allium* extract exhibited a higher final weight per rabbit and total weight compared to the control group, despite similar initial conditions. Furthermore, the biochemical analysis indicated no significant differences in most parameters, except for a statistical decrease in urea, cholesterol, and triglyceride levels, which remained within the normal range. Additionally, our investigation into the muscle tissue composition did not detect PTSO or its main metabolites in muscle, although novel metabolites were detected. Furthermore, changes in the alpha and beta diversity indexes of the microbiome, as well as significant alterations in the bacterial community composition of rabbits fed the *Allium*-based diet, were observed. These modifications could potentially correlate with enhanced intestinal integrity and improved nutrient utilization efficiency.

## Figures and Tables

**Table 1:** Diet composition for each group of control rabbits and rabbits fed with *Allium* extract during weaning and fattening.

	Weaning Diet (38 a 55 Days)		Fattening Diet (56 a 76 Days)	
Diet	Control	*Allium*	Control	*Allium*
**Ingredient (g/kg)**				
Dehydrated alfalfa meal	340.5	339.5	253	252
Wheat bran	190	190	240	240
Barley	120	120	160	160
Dried beet pulp	190	190	160	160
49% soybean meal	50	50	40	40
30% sunflower flour	70	70	100	100
Sunflower oil	10	10	15	15
Molasses	15	15	15	15
Calcium carbonate	1	1	4	4
Dicalcium phosphate	3.5	3.5	3	3
Sodium chloride	4	4	4	4
L-lysine HCl	1	1	1	1
DL-methionine	1	1	1	1
Vitamin and mineral premix	4	4	4	4
***Allium* extract** ^1^	--	1	--	1
Chemical composition (%)				
Dry matter	87.9	87.8	88.0	88.1
Crude protein	15.3	15.3	15.2	15.3
Ether extract	3.1	3.0	3.7	3.6
Crude fiber	16.6	16.7	15.1	15.2
Ash	6.8	6.8	6.6	6.7
Neutral detergent fiber	35.7	35.6	34.8	34.9
Acid detergent fiber	19.7	19.5	18.8	18.5
Acid detergent lignin	5.0	5.0	5.0	5.0
Starch	11.2	11.4	13.1	13.2

^1^ Standardized at 3% of PTSO.

**Table 2:** Effect of the use of *Allium* extract on the productive parameters of rabbits.

Item	Control (Mean)	Control (SD)	*Allium* Extract(Mean)	*Allium* Extract(SD)	*p* Value
Age at onset, day	38	-	38	-	-
No. of rabbits start	5571.25	90.59	5557.50	106.26	0.8504
Total weight, kg	5404.35	280.88	5056.68	165.38	0.3645
Starting weight, kg/rabbit	0.97	0.05	0.91	0.03	0.3645
Final age, day	77.00	0.71	75.63	1.49	0.4707
No. of rabbits end	5074.50	58.30	5271.25	97.62	0.1027
Final total weight, kg	12299.5	287.65	13742.47	328.96	0.0081
Final weight kg/rabbit	2.42	0.05	2.61	0.07	0.0406
Days in cycle, d	39.00	0.71	37.63	1.49	0.4707
No. of culls	496.75	65.85	286.25	28.28	0.01286
Mortality (%)	8.91	1.06	5.15	0.47	0.0084
Total gain per batch (TGB)	6895.24	89.44	8685.79	416.47	0.0026
ADG g/rabbit	37.28	0.76	45.11	1.35	0.0010
Average daily intake (ADI)	113.49	4.04	130.37	2.04	0.0045
Conversion rate	3.04	0.05	2.89	0.08	0.1040
Rabbits sacrificed	5009.50	60.42	5199.75	81.22	0.0816
Meat weight sacrificed (kg/rabbit)	1.37	0.03	1.48	0.03	0.0174
Total sacrificed meat weight	6875.81	144.02	7714.40	158.58	0.0037
% Sacrifice yield	56.64	0.81	56.91	0.48	0.8255

**Table 3:** Clinical biochemistry of male and female rabbits fed with control feed or *Allium* extract supplemented feed for 76 days. Values are mean ± SD for 10 rabbits/group. The difference between control and treated groups of rabbits was evaluated by unpaired t-test or Mann–Whitney test. The significance levels observed are *p < 0.05, **p < 0.01, or ***p < 0.001 in comparison to control group values.

Parameter	Control		*Allium* Extract	
	Mean	SD	Mean	SD
Total Protein (g/L)	61.40	6.26	58.40	4.30
Urea (mmol/L)	8.21	2.72	4.68 **	0.84
Creatinine (µmol/L)	78.00	27.05	90.70	16.28
Glucose (mmol/L)	6.99	0.85	6.88	0.44
Cholesterol (mmol/L)	2.88	0.62	1.25 **	0.23
Triglycerides (mmol/L)	2.72	1.23	0.66 ***	0.18
Total Billirubin (µmol/L)	<2.50	<2.50	<2.50	<2.50
Direct Bilirubin (µmol/L)	<1.50	<1.50	<1.50	<1.50
Indirect Bilirubin (µmol/L)	-	-	-	-
AST (U/L)	54.20	38.55	68.20	24.22
ALT (U/L)	51.00	20.06	61.60	13.65
ALP (U/L)	137.80	65.06	131.10	26.80
GGT (U/L)	7.30	2.91	6.70 *	1.42
Creatine Kinase (U/L)	424.88	349.16	379.90	169.58
Lactate dehydrogenase (U/L)	234.70	110.49	262.20	68.35
Ca (mmol/L)	3.06	0.17	3.59	0.15
P (mmol/L)	2.84	0.69	2.14 ***	0.14
Mg (mmol/L)	1.25	0.25	1.17	0.08
Na (mmol/L)	141.90	2.69	143.90	1.66
K (mmol/L)	5.97	1.00	6.09 *	0.44
Cl (mmol/L)	100.30	3.89	103.00	2.91
Fe (µg/dL)	125.70	49.77	148.90	40.66

ALT: alanine aminotransferase; ALP: alkaline phosphatase; AST: aspartate aminotransferase; GGT: gamma-glutamyl transferase.

**Table 4:** Analytical parameters calculated for control rabbit muscle samples spiked with different standards. Recovery estimations (%) at a concentration of 500 ppb, along with limits of detection (LOD) and quantification (LOQ) for PTSO, PTS, GSSP, and CSSP.

Compound	Linearity Range (ppb)	R^2^	Recovery (%)	LOD (ppb)	LOQ (ppb)
PTSO	50–1000	0.9991	106.83 ± 9.22	0.044	0.146
PTS	50–1000	0.9738	88.88 ± 9.57	0.246	0.821
GSSP	50–1000	0.9913	110.82 ± 16.52	2.782	9.272
CSSP	50–1000	0.9904	99.29 ± 9.85	2.175	7.251

**Table 5:** Metabolites found in muscles of rabbits dietary feed with *Allium* extract.

Metabolite ID	Formula	Parent Compound	Transformations	Composition Change	Δ Mass [ppm]	Calc. MW	RT [Min]
R1	C_6_ H_14_ O S_2_	PTSO	Dehydration, Reduction	−(O)	−1.13	166.04842	4.989
R2	C_6_ H_10_ O_2_ S_2_	PTSO	Desaturation, Desaturation	−(H_4_)	−0.41	178.01215	3.518
R3	C_6_ H_10_ O_2_ S_2_	PTSO	Desaturation, Desaturation	−(H_4_)	−0.24	178.01218	3.464
R4	C_11_ H_26_ N_2_ O S_2_	PTSO	Nitro Reduction, Ornitine Conjugation	−(O) + (C_5_ H_12_ N_2_)	1.61	266.14908	5.124
R5	C_8_ H_21_ N O S_2_	PTSO	Nitro Reduction, Reduction, Glycine Conjugation	−(O) + (C_2_ H_7_ N)	−1.41	211.10616	4.983
R6	C_11_ H_24_ N_2_ O S_2_	PTSO	Desaturation, Nitro Reduction, Ornitine Conjugation	−(O) + (C_5_ H_10_ N_2_)	1.36	264.13336	5.446
R7	C_11_ H_26_ N_2_ O S_2_	PTSO	Nitro Reduction, Ornitine Conjugation	−(O) + (C_5_ H_12_ N_2_)	1.46	266.14904	5.346
R8	C_11_ H_20_ N_2_ O_4_ S_2_	PTSO	Desaturation, Glutamine Conjugation	+(C_5_ H_6_ N_2_ O_2_)	2.46	308.08721	0.782
R9	C_6_ H_14_ S	PTSO	Dehydration, Nitro Reduction, Thiourea to Urea	−(O_2_ S)	2.75	118.08195	0.633

MW: molecular weight; RT: retention time.

## Data Availability

The original contributions presented in the study are included in the article/supplementary material, further inquiries can be directed to the corresponding author.

## Supplementary Materials

The following supporting information can be downloaded at: https://www.mdpi.com/article/10.3390/foods13233976/s1, Figure S1. Representative chromatogram showing the detection of PTSO in the analyzed feed sample.
