# Supplementary material for: Effects of a Diet of Allium Extract on Growth, Biochemistry, Metabolism, and Gut Microbiota of Rabbits (Oryctolagus cuniculus)
Source: Foods. 2024 Dec 9;13(23):3976. doi: 10.3390/foods13233976 (PMC11641707; doi:10.3390/foods13233976)
Supplement: Supplementary file 1 [file foods-13-03976-s001.zip › foods-3341433-supplementary.docx]

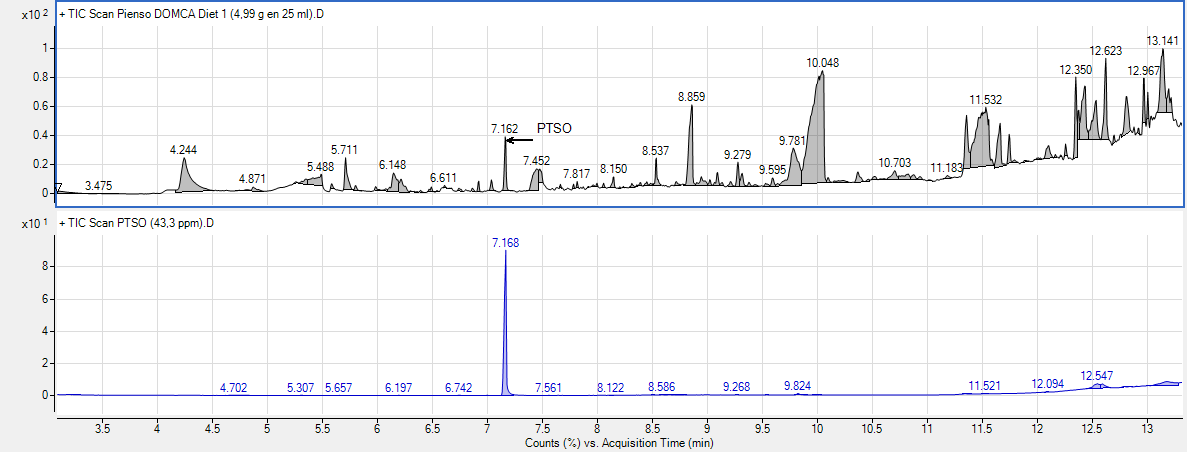


**Figure S1.** Representative chromatogram showing the detection of PTSO in the analyzed feed sample.
